# Supplementary material for: ADAM17‐triggered TNF signalling protects the ageing Drosophila retina from lipid droplet‐mediated degeneration
Source: EMBO J. 2020 Jul 26;39(17):e104415. doi: 10.15252/embj.2020104415 (PMC7459420; doi:10.15252/embj.2020104415)
Supplement: Supplementary file 1 — Appendix [file EMBJ-39-e104415-s001.pdf]

Appendix:

Appendix Figure S1: Normal eye development in ADAM17$^{-/-}$ mutant and characterisation of LDs

Appendix Figure S2: Age-dependent clearance of LD in ADAM17 mutant retinas

Appendix Figure S3: Sequence comparison and AP-shedding assays of Drosophila ADAM17

Appendix Figure S4: LD counts and MDA amounts for iPSC microglia cells treated with inhibitors for ADAM17 and/or ADAM10

Supplemental fly strains

Appendix Figure S1

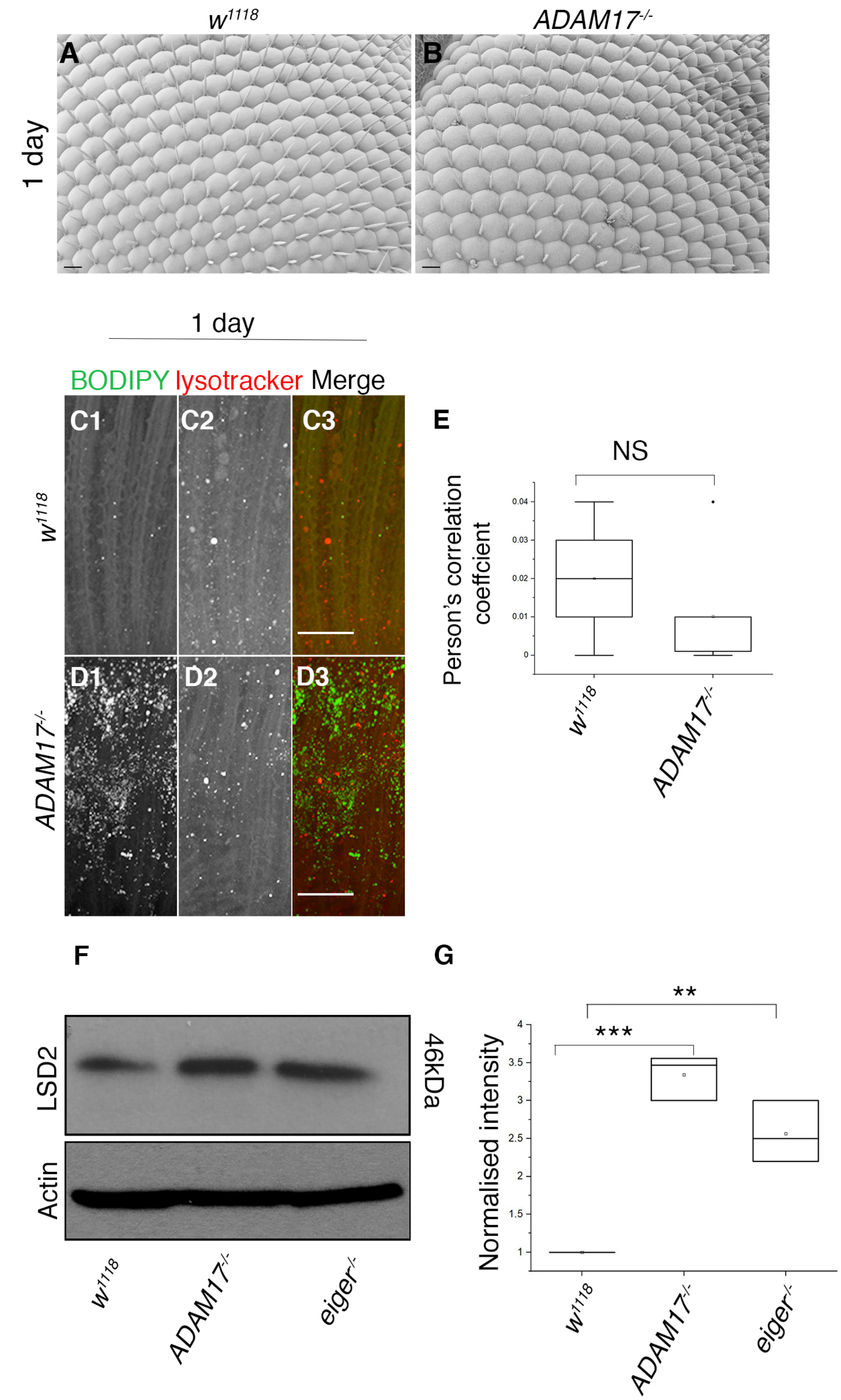

Appendix Figure S1: Normal eye development in *ADAM17*$^{-/-}$ mutant and characterisation of LDs

A-B. Scanning Electron Microscopy (SEM) images of the surface of adult retinas corresponding to (A) wild type; and (B) *ADAM17*$^{-/-}$; n=3 flies for each. Scale bar: 10μm.

C-D. Co-labelling of (C) wild type; and (D) *ADAM17*$^{-/-}$ retinas with BODIPY and lysotracker; n=10 flies for each.

E. Comparison of Pearson's correlation coefficient for BODIPY and lysotracker labelling in the genotypes mentioned above. Box end points represent the upper (75%) and lower (25%) quartiles, whiskers define the maximum 95th Percentile and minimum 5th Percentile values respectively, central band is the median, square is the mean and diamond an outlier.

F-G. Analysis of LSD2 protein levels in head lysates of wild type, *ADAM17*$^{-/-}$ and *eiger*$^{-/-}$ head lysates using western blot; n=3 biological replicates. Scale bar :10μm Box end points represent the maximum and minimum values respectively, central band is the median and square is the mean.

Data Information: All datasets were quantified for significance using Student's t test. ***p<.001, **p<.01.

Appendix Figure S2

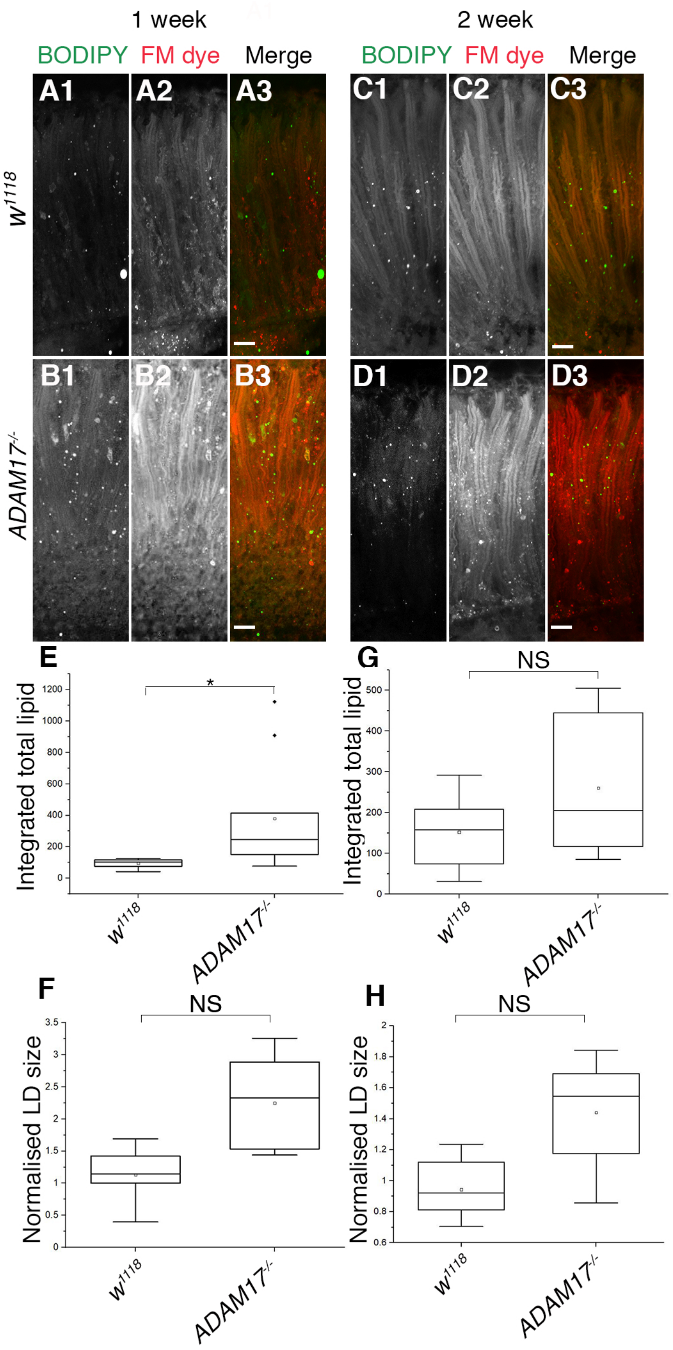

Figure S2: Age-dependent clearance of LD in ADAM17 mutant retinas

A-D. Fluorescent images of 1week old (A) wild type and (B) *ADAM17*$^{-/-}$; 2 week old (C) wild type and (D) *ADAM17*$^{-/-}$ mutant retinas labelled with BODIPY (green) and FM dye (red).

E-H. Quantitation of BODIPY staining (shown in A-D), depicted as integrated total lipid (E, G) and normalised lipid droplet size (F, H); n=10 for each genotype. Box end points represent the upper (75%) and lower (25%) quartiles, whiskers define the maximum 95th Percentile and minimum 5th Percentile values respectively, central band is the median, square is the mean and diamond an outlier.

Data Information: Normality and homogeneity of variance were used to determine whether the data met the assumptions of the statistical test used. All datasets were assumed to be independent. Datasets with unequal variance were analysed using the Kruskal Wallis test followed by Dunn's test for post hoc analysis for significance due to unequal sample sizes. All other datasets were quantified for significance using Student's t test.

*p<.05

Appendix Figure S3

A

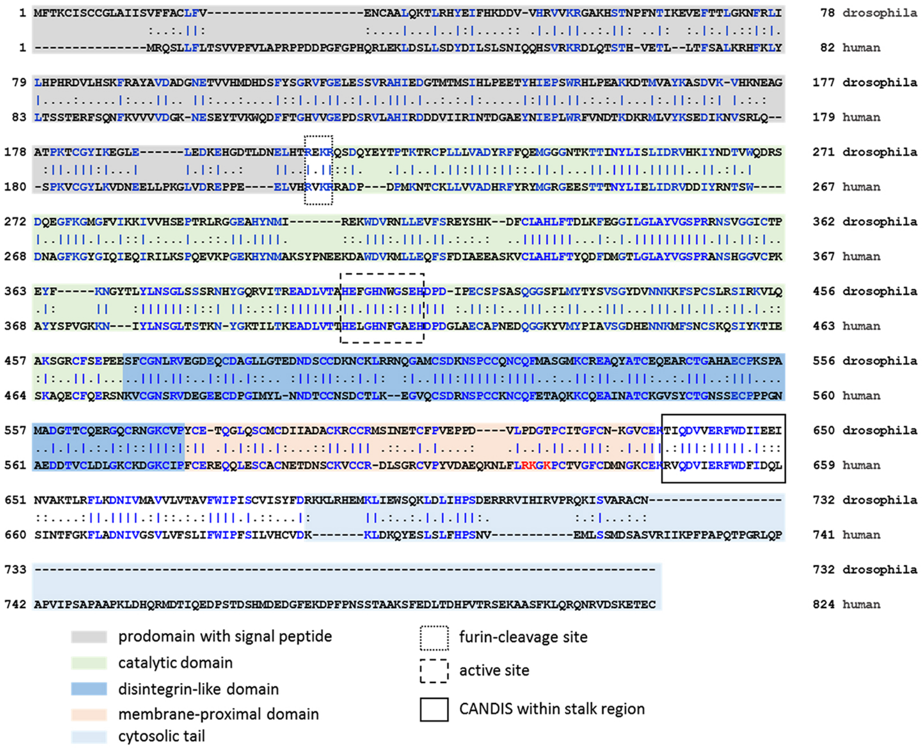

B

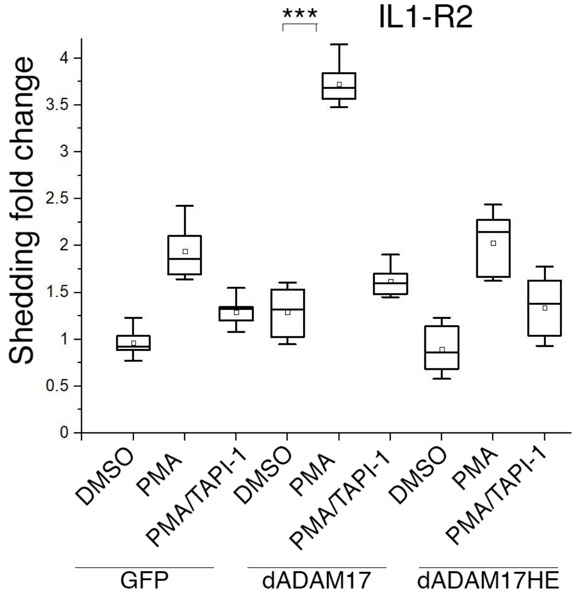

Appendix Figure S3: Sequence comparison and AP-shedding assays of Drosophila ADAM17

A. Sequence alignment of Drosophila ADAM17 with its human counterpart.

B. AP-shedding assays performed for IL1-R2 in HEK cells, lacking both human ADAM17 and ADAM10, with either GFP control, full length Drosophila ADAM17, or an activity dead mutant of Drosophila ADAM17, in the presence of either PMA or PMA and TAPI-1 (DMSO is used as control); n=10 for each condition. Box end points represent the upper (75%) and lower (25%) quartiles, whiskers define the maximum 95th Percentile and minimum 5th Percentile values respectively, central band is the median, square is the mean and diamond an outlier.

Data Information: All datasets were quantified for significance using Student's t test. ***p<.001

Appendix Figure S4

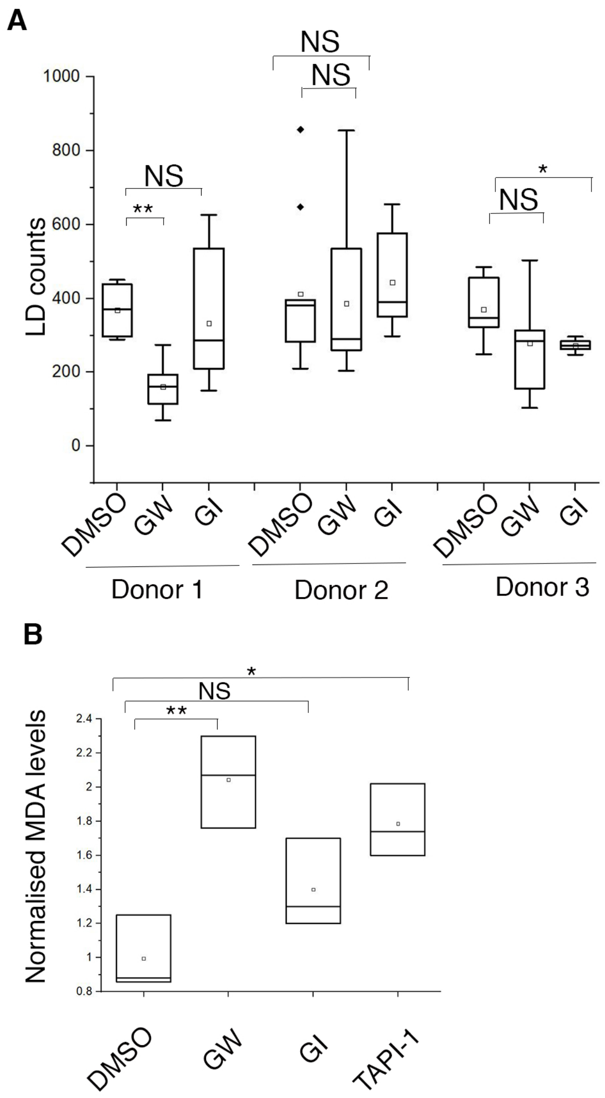

Appendix Figure S4: LD counts and MDA amounts for iPSC microglia cells treated with inhibitors for ADAM17 and/or ADAM10

A. LD counts for iPSC derived microglia-like cells from 3 different donors treated with either DMSO, GW or GI for a period of 24hours; n=10 cells for each treatment, per donor. Box end points represent the upper (75%) and lower (25%) quartiles, whiskers define the maximum 95th Percentile and minimum 5th Percentile values respectively, central band is the median, square is the mean and diamond an outlier.

B. Levels of Malondialdehyde (normalised to DMSO controls) in cell lysates from Donor No.2 treated with either DMSO, GW, GI or TAPI-1 for 24 hours; n=3 biological replicates. Box end points represent the maximum and minimum values respectively, central band is the median and square is the mean,

Data Information: All datasets were quantified for significance using Student's t test. **p<.01, *p<.05

Supplemental fly genotypes:

Fig. S1:

w^{1118}

;+/+;ADAM17$^{-/-}$

spa-GAL4;+/+;*ADAM17$^{-/-}$*

spa-GAL4;UAS-ADAM17-WT/+;

spa-GAL4;UAS-ADAM17-WT/+;*ADAM17$^{-/-}$*

Fig.S3:

spa-GAL4;+/+;*UAS-LacZ/+*

spa-GAL4;+/+;*UAS-$eiger_i$/+*

spa-GAL4;+/+;*UAS-$grnd_i$/+*

spa-GAL4;+/+;*UAS-wgn_i/+*

;+/+;*Actin-GAL4*/*UAS-LacZ*

+/+;;Actin-GAL4/*UAS-$eiger_i$*

;+/+;Actin-GAL4/*UAS-grdni*

;+/+;Actin-GAL4/*UAS-wgn_i*

;elav-GAL4/+;*UAS-LacZ/+*

;elav-GAL4/+;*UAS-egr_i/+*

;elav-GAL4/+;*UAS-$grnd_i$/+*

w^{1118}

$egr^{-/-}$

$grnd^{-/-}$
