# Supplementary material for: ADAM17‐triggered TNF signalling protects the ageing Drosophila retina from lipid droplet‐mediated degeneration
Source: EMBO J. 2020 Jul 26;39(17):e104415. doi: 10.15252/embj.2020104415 (PMC7459420; doi:10.15252/embj.2020104415)
Supplement: Supplementary file 2 — Expanded View Figures PDF [file EMBJ-39-e104415-s002.pdf]

Expanded View Figures

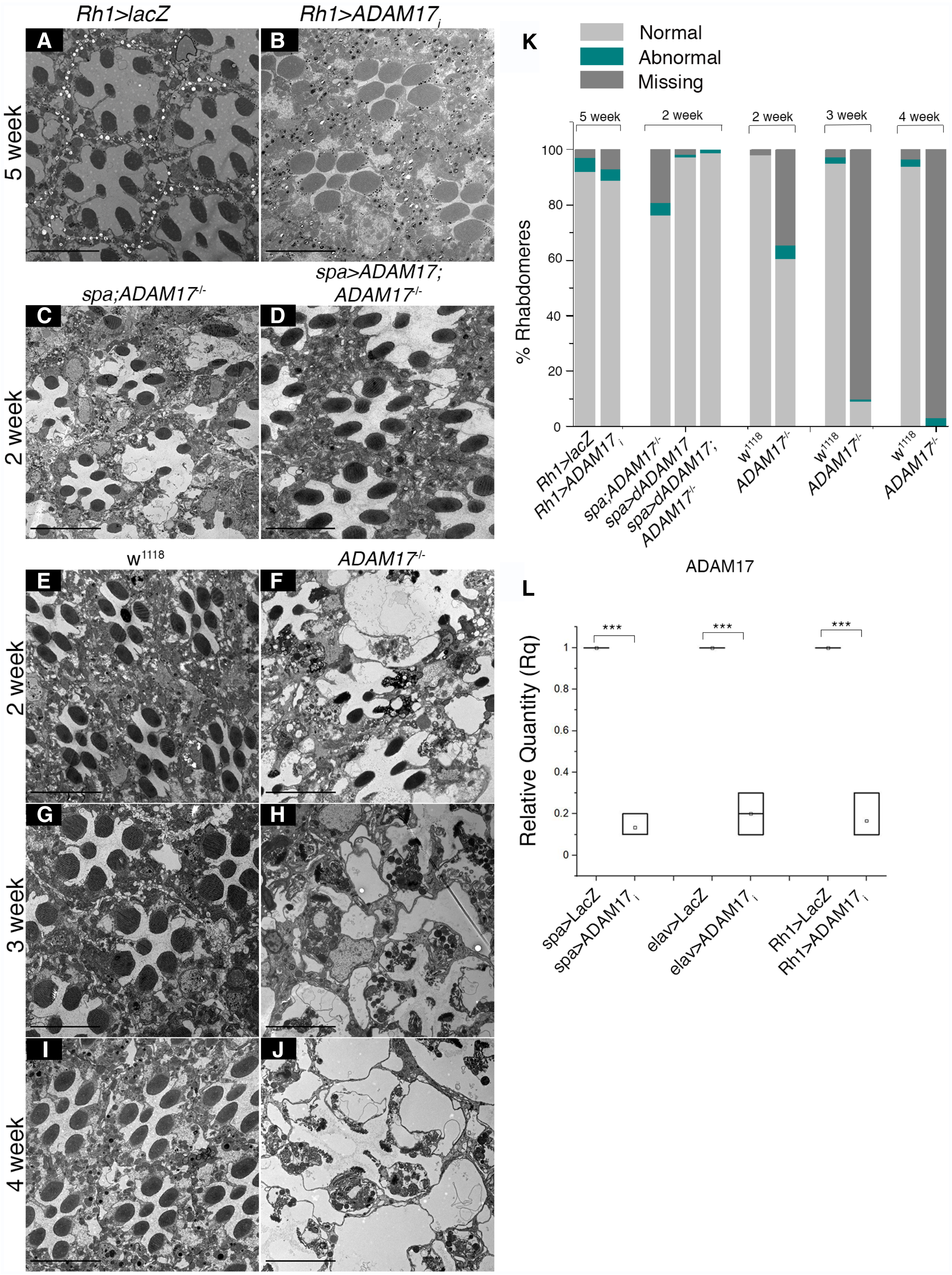

Figure EV1.

Figure EV1. Loss of ADAM17 in PGCs induces age-dependent degeneration.

A–J TEM images of adult retinas; (A) overexpressing lacZ in neurons; (B) RNAi for ADAM17 in neurons; (C) ADAM17$^{-/-}$ mutant; (D) *ADAM17*$^{-/-}$ mutant overexpressing WT-ADAM17 under control of the *spa-Gal4* driver (*spa>ADAM17*) in PGCs; (E, G, I) wild type; or (F, H, J) *ADAM17*$^{-/-}$ mutant retinas at 2 weeks (E, F), 3 weeks (G, H) and 4 weeks (I, J).

K Quantitation of the numbers of normal, abnormal and missing rhabdomeres from the TEM images corresponding to the genotypes mentioned above; n = 180 ommatidia from 3 different fly retinas for each.

L qPCR measurements of knockdown efficiency of ADAM17 with *sparkling*, *elav* or *Rh1* GAL4s; n = 3 independent biological replicates with 3 technical replicates for each experiment. n = 3 biological replicates, with 3 technical replicates for each genotype. The box end points represent the maximum and minimum values respectively, the central band is the median, and the square is the mean.

Data information: All data were quantified for significance using Student's t test. ***$P < 0.001$. Scale bars: 10 μm.

© 2020 The Authors

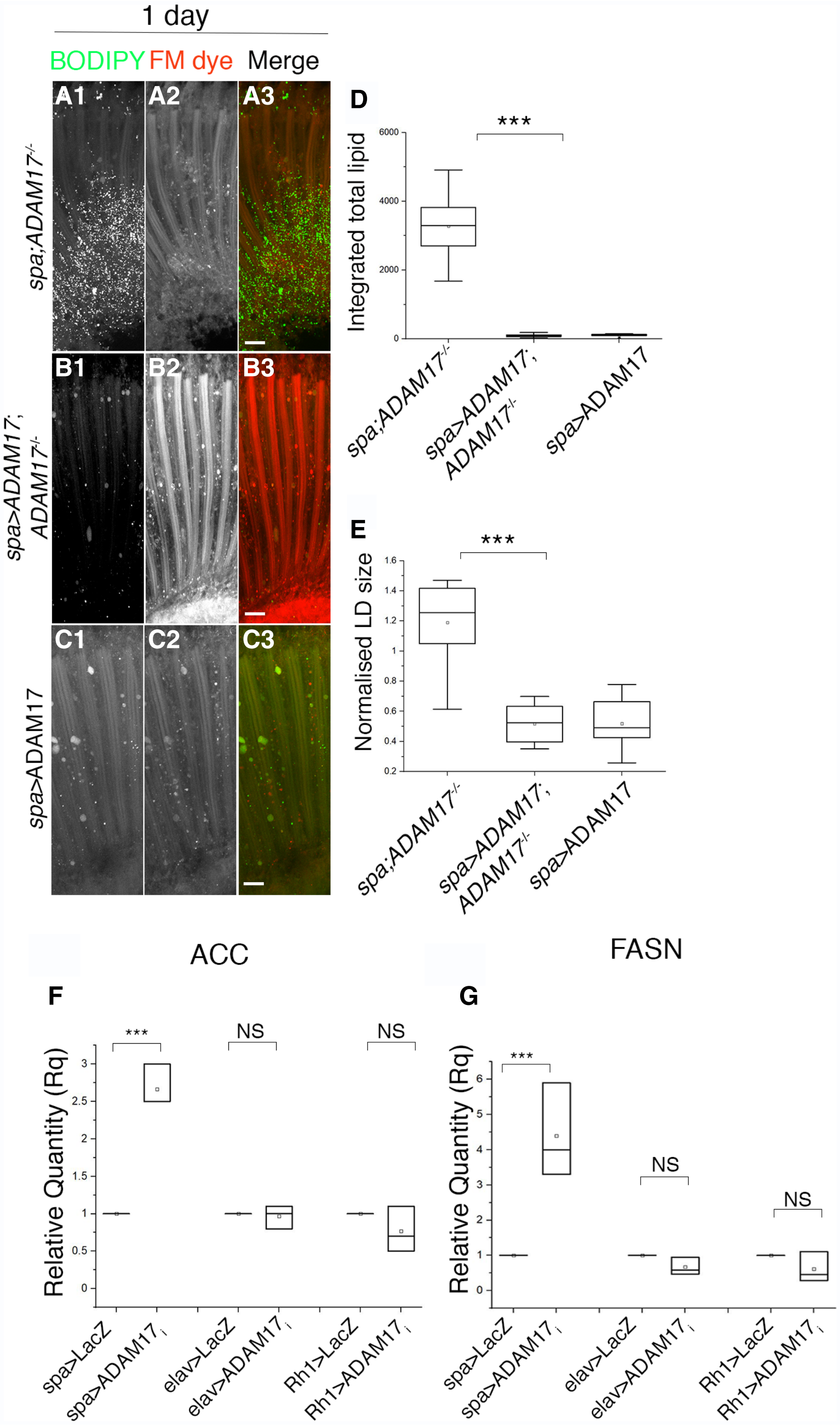

Figure EV2. Rescue of LD phenotype with WT-ADAM17 and expression of lipogenic genes with a knockdown of ADAM17 using different drivers.

A–C Fluorescent images of 1-day-old retinas, stained with BODIPY (green) and FM dye (red); (A) *ADAM17*$^{-/-}$ mutant; (B) glial-specific overexpression of ADAM17 in *ADAM17*$^{-/-}$ mutant; or (C) glial-specific overexpression of ADAM17 in a wild-type background. Scale bars: 10 µm.

D, E Quantitation of BODIPY staining (shown in A–C) depicted as (D) integrated total lipid and (E) normalised lipid droplet size; n = 10 for each genotype. The box end points are the upper (75%) and lower (25%) quartiles, the whiskers define the maximum 95th percentile and minimum 5th percentile values, respectively, the central band is the median, and the square is the mean.

F, G qPCR measurements of lipogenic transcripts of ACC and FASN across different knockdowns of ADAM17 with *sparkling, elav or Rh1* GAL4s; n = 3 biological replicates, with 3 technical replicates for each genotype. The box end points represent the maximum and minimum values, respectively, the central band is the median, and the square is the mean.

Data information: All data were quantified for significance using Student's t test. ***$P < 0.001$.

Figure EV3. Loss of ADAM17 does not affect LD in larval and pupal tissues.

A–F Fluorescent images of either wild-type or *ADAM17*$^{-/-}$ mutant larval (A, B) brain; (C, D) eye imaginal disc; and (E, F) wing imaginal disc, stained with BODIPY (green) and FM dye (red).

G Quantitation of BODIPY staining (shown in A–F) depicted as integrated total lipid; n = 10 for each genotype. The box end points represent the maximum and minimum values, respectively, the central band is the median, and the square is the mean. Data were analysed using the Kruskal–Wallis test followed by Dunn's test for *post hoc* analysis for significance due to unequal sample sizes.

H FASN mRNA transcript levels measured by qPCR in wild-type and *ADAM17*$^{-/-}$ larvae; n = 4 biological replicates, with 3 technical replicates for each genotype. The box end points are the upper (75%) and lower (25%) quartiles, the whiskers define the maximum 95th percentile and minimum 5th percentile values, respectively, the central band is the median, and the square is the mean.

I, J Fluorescent images of either wild-type (I) or *ADAM17*$^{-/-}$ (J) mutant pupal (I, J) retinas stained with BODIPY (green) and FM dye (red).

K Quantitation of BODIPY staining (shown in A–F) depicted as integrated total lipid; n = 10 for each genotype. The box end points are the upper (75%) and lower (25%) quartiles, the whiskers define the maximum 95th percentile and minimum 5th percentile values, respectively, the central band is the median, and the square is the mean.

L Comparison of mRNA levels of ADAM17 between pupae and 1-day-old wild-type adults. n = 3 biological replicates, with 3 technical replicates for each genotype. The box end points represent the maximum and minimum values, respectively, the central band is the median, and the square is the mean.

M Whole-mount pupal retina stained with anti-ADAM17 (green) and FM dye to mark the photoreceptor membranes (red); n = 10 fly retinas.

Data information: Data were quantified for significance using Student's t test. ***$P < 0.001$. Scale bars: 10 μm.

© 2020 The Authors

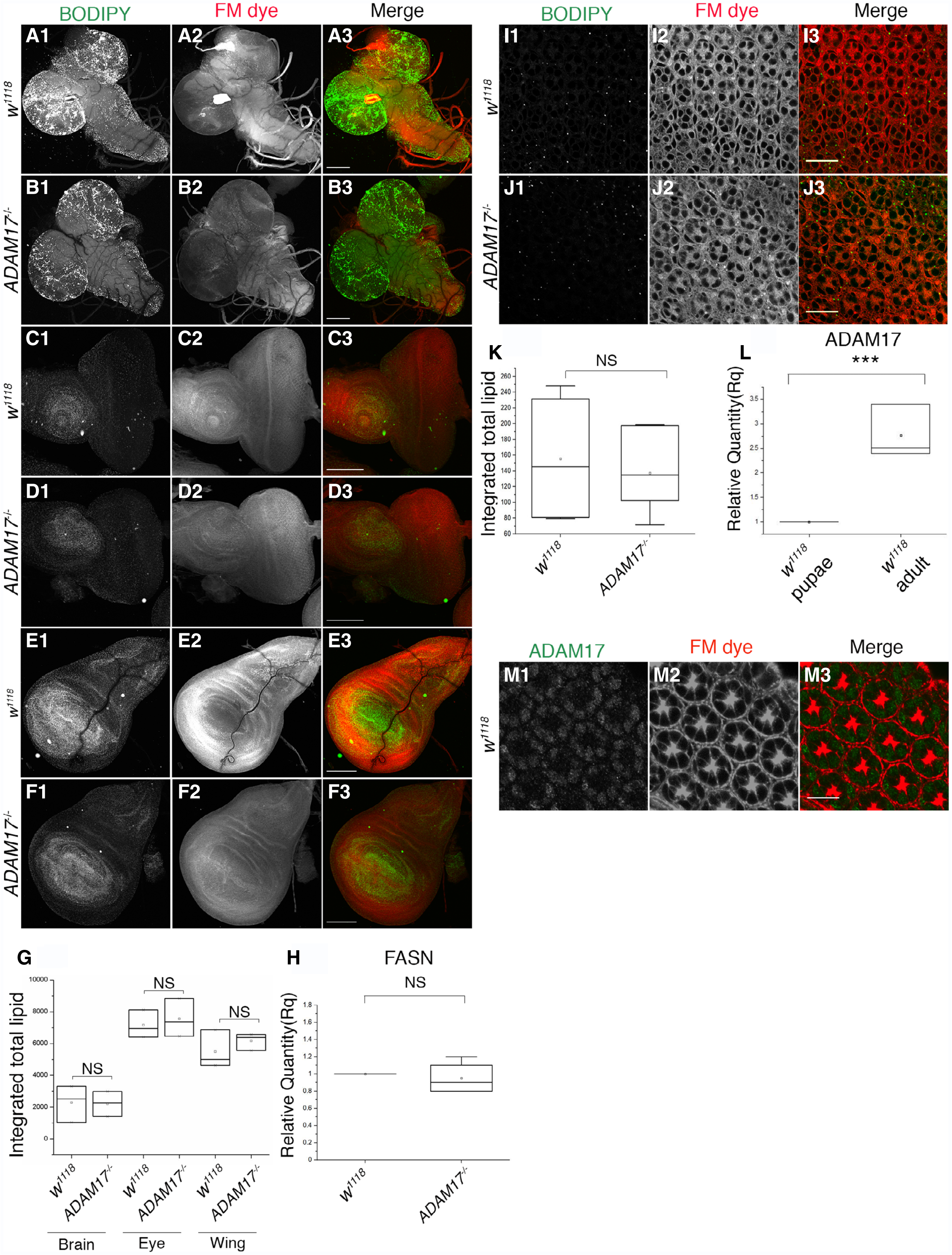
BODIPY
FM dye
Merge
A1
A2
A3
B1
B2
B3
C1
C2
C3
D1
D2
D3
E1
E2
E3
F1
F2
F3
w1118
ADAM17-/-
I1
I2
I3
J1
J2
J3
K
NS
Integrated total lipid
L
ADAM17
***
Relative Quantity(Rq)
pupae
adult
ADAM17
M1
M2
M3
G
NS
Brain
Eye
Wing
H
FASN

Figure EV3.

Figure EV4. Loss of eiger and grindelwald, but not wengen specifically in PGC leads to abnormal LD accumulation and age-associated retinal degeneration.

A–D Fluorescent images of 1-week-old retinas labelled with BODIPY (green) and FM7 dye (red); overexpressing (A) *lacZ*; (B) *eiger* RNAi; (C) *grindelwald* RNAi or (D) *wengen* RNAi specifically in PGC; $n = 10$ for each genotype.

E Quantitation of BODIPY staining (observed in A–D) shown as integrated total lipid count. The box end points are the upper (75%) and lower (25%) quartiles, the whiskers define the maximum 95th percentile and minimum 5th percentile values, respectively, the central band is the median, and the square is the mean.

F, G Quantitation of BODIPY staining shown as integrated total lipid count in retinas overexpressing *lacZ*, *eiger* RNAi, *grindelwald* RNAi or *wengen* RNAi either throughout the retina (F); and the neurons (G); $n = 10$ for each (fluorescent images not shown). The box end points are the upper (75%) and lower (25%) quartiles, the whiskers define the maximum 95th percentile and minimum 5th percentile values, respectively, the central band is the median, and the square is the mean.

H, I mRNA transcript levels of ACC and FASN measured by qPCR in retinas expressing *lacZ*, *eiger* RNAi, *grindelwald* RNAi in PGC; $n = 3$ independent biological replicates with 3 technical replicates for each experiment. $n = 3$ biological replicates, with 3 technical replicates for each genotype. The box end points represent the maximum and minimum values, respectively, the central band is the median, and the square is the mean.

J–L TEM images of 1-day-old retinas expressing (J) *lacZ*; (K) *eiger* RNAi; (L) and *grindelwald* RNAi specifically in neurons.

M–O TEM images of 5-week-old retinas of (M) wild type; (N) *eiger;* and (O) *grindelwald* mutants.

P Quantitation of the numbers of normal, abnormal and missing rhabdomeres observed in the TEM images corresponding to the genotypes mentioned above in M–O; $n = 180$ ommatidia from 3 different fly retinas for each.

Data information: Data were quantified for significance using Student's *t* test. ***$P < 0.001$, **$P < 0.01$, *$P < 0.05$. Scale bars: 10 μm.

© 2020 The Authors

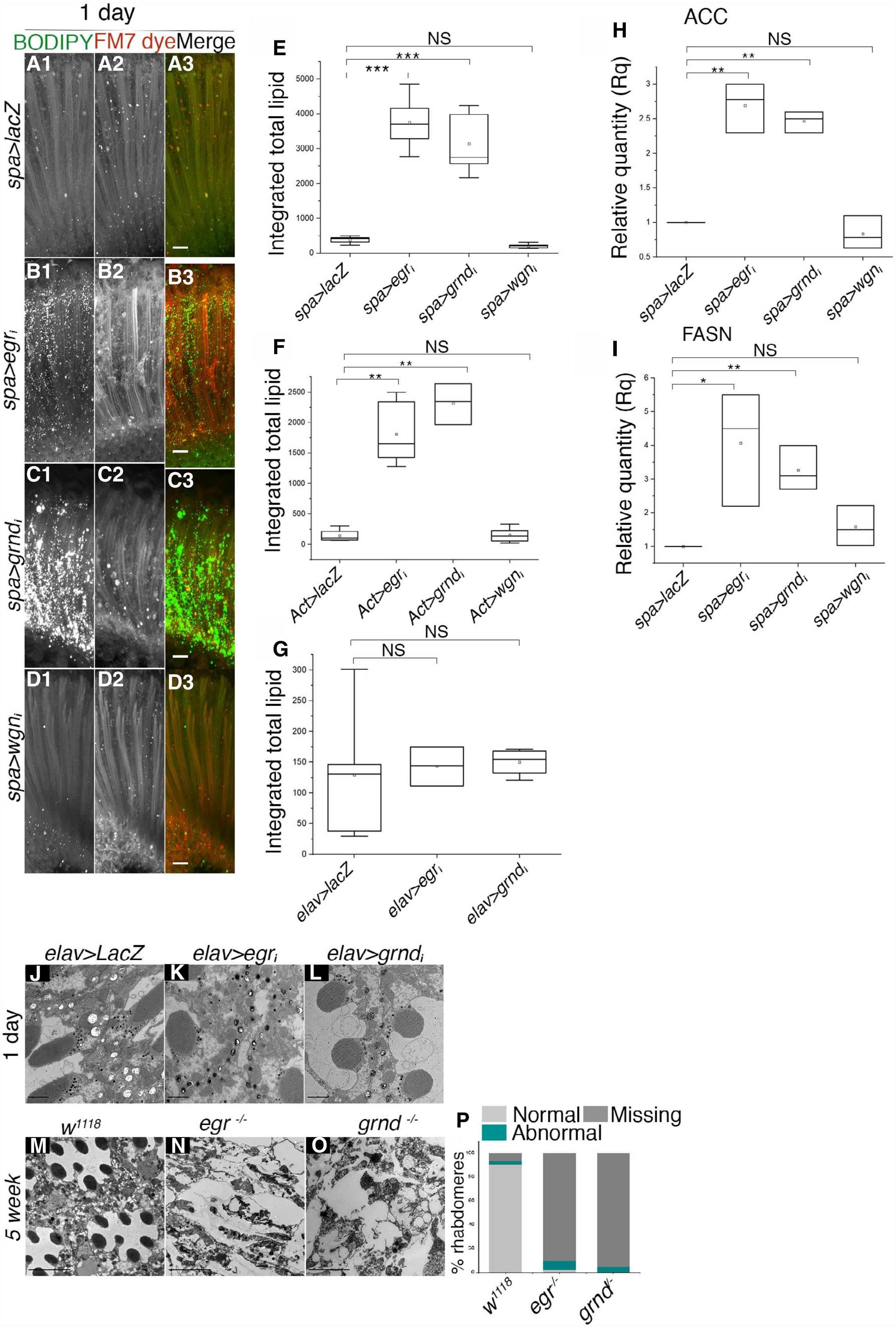
1 day
BODIPY FM7 dye Merge
A1 A2 A3
spa>lacZ
B1 B2 B3
spa>egr$_i$
C1 C2 C3
spa>grnd$_i$
D1 D2 D3
spa>wgn$_i$
E
Integrated total lipid
NS
***
spa>lacZ
spa>egr$_i$
spa>grnd$_i$
spa>wgn$_i$
F
Act>lacZ
Act>egr$_i$
Act>grnd$_i$
Act>wgn$_i$
G
elav>lacZ
elav>egr$_i$
elav>grnd$_i$
H
ACC
Relative quantity (Rq)
I
FASN
elav>LacZ
elav>egr$_i$
elav>grnd$_i$
J K L
1 day
w^{1118}
$egr^{-/-}$
$grnd^{-/-}$
M N O
5 week
P
Normal
Missing
Abnormal
% rhabdomeres

Figure EV4.

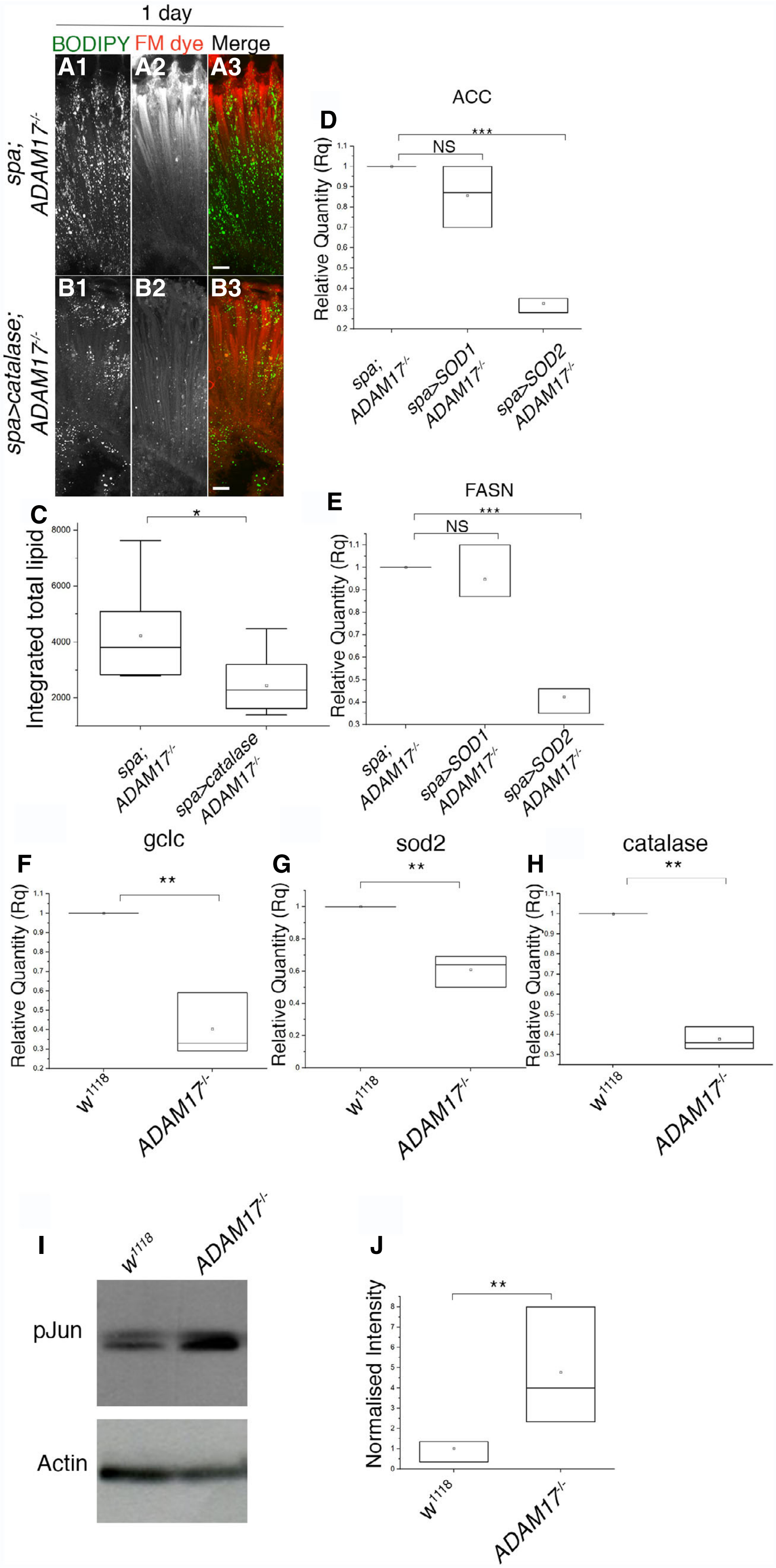

Figure EV5. Lipid droplet counts and lipogenic transcript levels in glial-specific overexpression of catalase, SOD1 or SOD2.

A, B Fluorescent images of retinas stained with BODIPY (green) and FM dye (red) corresponding to (A) *ADAM17*$^{-/-}$; and (B) glial-specific overexpression of catalase in *ADAM17*$^{-/-}$ background; n = 10 for each genotype. Scale bars: 10 µm.

C Quantitation of BODIPY staining (shown in A and B), depicted as integrated total lipid. The box end points are the upper (75%) and lower (25%) quartiles, the whiskers define the maximum 95th percentile and minimum 5th percentile values, respectively, the central band is the median, and the square is the mean.

D, E qPCR measurement of mRNA transcript levels of ACC and FASN from *ADAM17*$^{-/-}$ retinas and retinas overexpressing either SOD1 or SOD2 within glial cells in an *ADAM17*$^{-/-}$ background. n = 3 biological replicates, with 3 technical replicates for each genotype. The box end points represent the maximum and minimum values, respectively, the central band is the median, and the square is the mean.

F–H mRNA transcript levels of gclc, sod2 and catalase measured by qPCR in wild-type and *ADAM17*$^{-/-}$ retinas; n = 3 biological replicates, with 3 technical replicates for each genotype. The box end points represent the maximum and minimum values, respectively, the central band is the median, and the square is the mean.

I Western blot analysis of phosphorylated Jun (pJun) and actin levels from head lysates of wild-type and *ADAM17*$^{-/-}$mutants.

J Averaged intensity levels of pJun normalised to actin, as inferred from Western blots. n = 3 biological replicates, with 3 technical replicates for each genotype. The box end points represent the maximum and minimum values, respectively, the central band is the median, and the square is the mean.

Data information: Data were quantified for significance using Student's t test. ***$P < 0.001$, **$P < 0.01$, *$P < 0.05$.

 © 2020 The Authors
